# Supplementary material for: Predicting invasive mechanical ventilation in COVID 19 patients: A validation study
Source: PLoS One. 2024 Jan 2;19(1):e0296386. doi: 10.1371/journal.pone.0296386 (PMC10760863; doi:10.1371/journal.pone.0296386)
Supplement: S1 File — (DOCX) [file pone.0296386.s001.docx]

**Supplement 1. Predicted Risk Level calculation**

To estimate the risk of deterioration we introduced a special coefficient, designed to mimic clinical considerations, yet calculated from the XGboost results:

- 1. Risk level which is lower from the threshold is considered “negative”.
  2. Risk level greater than the threshold represents a risk of intubation. However, as the precision of values which are close to the threshold is low, this estimation should be considered cautiously from clinical perspective.
  3. Very high-risk levels (much greater than the threshold) are relatively rare, yet they are highly important, as they reflect great risk for intubation. These levels shall be considered as the end of the tail of the distribution.
  4. The risk level shall be intuitive, to reflect the chance of a patient to get IMV. Therefore the coefficient was designed on a scale of 0 to 1. Any result greater than 0 should reflect a positive prediction to intubation, yet lower numbers should be considered with a matter of inaccuracy.
  5. The histogram above the threshold can be approximated with uniform distribution.
  6. Scale and threshold T are different between different models, and among different runs of each model. Hence, the risk level is scaled independently by the formula presented above.

Therefore, we defined the coefficient:

$c_{f}(i)=\left\{ \begin{matrix} 0, & if patient i is "negative" \\ \frac{\min\left( p_{i},p_{f}^{max} \right) - T}{p_{f}^{max} - T}, & if patient i is "positive" \end{matrix} \right.$,

Where:

*i* is patient index;

*T* is the threshold obtained by the “Choose Threshold” procedure, see below;

$p_{i}$ is the probability of patient *i* to have a positive result by the model;

*f* is the fraction of distribution above the threshold; to define its tail we use *f*=0.95;

$p_{f}^{max}$ is *f* ( *f*=0.95) level quantile of the distribution above the threshold.

Procedure “Choose Threshold”

Threshold *T* is chosen as follows:

1. Choose point on ROC with False Positive Rate (FP) = 0.2;
2. If True Positive Rate (TP) corresponding to it is greater than 0.95 then
   1. find on ROC the point with TP=0.95
   2. find FP corresponds to TP=0.95
3. find threshold *T* corresponds to the chosen pair TP/FP.

An example of histogram of probabilities that are output from XGBoost testing presented on Figure S1-1.

***Number of points***

………………………

………………………

**Positives**

**Negatives**

**Threshold *T***

***Probability Scale***

Figure S1-1. Output of XGBoost testing, showing Histogram of probabilities of ventilation. Intuitively it can be interpreted as a likelihood for a patient to get IMV.
