# Supplementary material for: Predicting invasive mechanical ventilation in COVID 19 patients: A validation study
Source: PLoS One. 2024 Jan 2;19(1):e0296386. doi: 10.1371/journal.pone.0296386 (PMC10760863; doi:10.1371/journal.pone.0296386)
Supplement: S2 File — (DOCX) [file pone.0296386.s002.docx]

**Supplement 2. Strong zone formula**

High risk for an event is a situation in which the prediction is consistently high. This can be achieved, conceptually, in two ways: (1) An extremely high risk for a short period of time, or (2) a high risk (not necessarily “extremely high”) for a longer time. Therefore, criteria for this category, should account the “average risk” of a patient. Obviously, the longer time the risk itself is high, the importance of the average risk decreases.

Based on it, we constructed the following formal criteria for strong zone prediction of intubation:

1. Patients who had only one hourly prediction, which was positive. These patients were observed for very short period of time before intubation or mortality; and/or
2. At least one prediction risk equals to 1; or
3. At least 3 hours with risk level equal or greater than 0.8, **and**
4. Time with average risk level is greater than 0.5 divided by number of hours when risk level is equal or greater than 0.8. This is calculated based on the following formula:

$$r_{avg}>\frac{0.5}{N_{r\geq0.8}}$$

where

$r_{avg}$ is the average risk level, $r_{avg}=\frac{\sum_{t=1}^{N} r_{t}}{N}$,

*N* is number of hours observed for this patient,

$r_{t}$ is risk at *t*-th hour,

$N_{r\geq0.8}$ is number of hours when the risk was not less than 0.8.
