# Supplementary material for: Predicting invasive mechanical ventilation in COVID 19 patients: A validation study
Source: PLoS One. 2024 Jan 2;19(1):e0296386. doi: 10.1371/journal.pone.0296386 (PMC10760863; doi:10.1371/journal.pone.0296386)
Supplement: Attachment — Submitted filename: Rebuttal letter FINAL2.docx [file pone.0296386.s003.docx]

**Rebuttal letter**

Dear Editor,

We wish to thank you and the reviewers for the comments. After editing the manuscript accordingly, we feel it is indeed better reflects our work. Below is a detailed response to each of the comments. It is formatted in blue.

A native English speaker edited and corrected the manuscript.

Journal Requirements

Please ensure that your manuscript meets PLOS ONE's style requirements, including those for file naming. The PLOS ONE style templates can be found at https://journals.plos.org/plosone/s/file?id=wjVg/PLOSOne_formatting_sample_main_body.pdf

and

https://journals.plos.org/plosone/s/file?id=ba62/PLOSOne_formatting_sample_title_authors_affiliations.pdf

Headings, tables, figures and affiliations were symbolled based on PLOS ONE’s style requirements.

Journal Requirements & Reviewer #1

I would just really encourage the research team to make their deidentified data publicly available in order to further strengthen their methodological claims and allow for the generation of further studies and algorithms by the scientific community.

Important: If there are ethical or legal restrictions to sharing your data publicly, please explain these restrictions in detail. Please see our guidelines for more information on what we consider unacceptable restrictions to publicly sharing data: http://journals.plos.org/plosone/s/data-availability#loc-unacceptable-data-access-restrictions. Note that it is not acceptable for the authors to be the sole named individuals responsible for ensuring data access.

Rabin Medical Center is part of Clalit Health Services, the biggest HMO service provider in Israel. Clalit Health Service policy doesn’t allow sharing of patients’ data even if it is de-identified. However, it will be possible to share the data upon a reasonable request, either to the corresponding author or to Mis. Hagit Hendel, CIO of Rabin Medical Center, available at hagithe1@clalit.org.il.

Reviewer #2

Primarily I recommend that the authors change the bibliography entry style to the style required by the journal, to be retrieved in the instructions for authors.

https://journals.plos.org/plosone/s/submission-guidelines or perhaps use an automatic bibliography creator for proper bibliography style selection.

The reference list style was changed to Vancouver.

I also advise authors to correct typos and grammatical errors that are present and repeated in the text, perhaps even having the work seen by a native speaker who can adjust and make the manuscript fluid.

A native English speaker has edited the manuscript.

Keyword.

1. First, I advise authors to include keywords in the main manuscript as well, so that when downloading the work, they are found and evaluated, thus slightly facilitating the work of reviewers as well.

2. As for the keywords present it can be said that they are all appropriate for the work done but unfortunately none, except for one, present in the MESH database. (https://www.ncbi.nlm.nih.gov/mesh/)

I recommend a thorough review and correlation with the MESH database.

Below are some suggestions:

- Machine learning tool  machine learning, Algorithms, Artificial Intelligence

- Invasive mechanical ventilation prediction  mechanical ventilations, Probability Learning,

- COIVD- 19  spelling to be corrected, perhaps include additional keywords, perhaps more generic, e.g., ARDS, Respiratory Distress Syndrome, SARS-CoV-2

Keywords were added to the main manuscript, and changed as suggested (including typos correction), as follows:

machine learning; Algorithms; mechanical ventilation; probability learning; COVID-19; SARS-CoV-2; ARDS

Introduction

3. Line 29 The authors state the treatment of respiratory failure, but then cite 2 articles referring to noninvasive ventilation, one of which is recent, instead one quite dated. In this context, it would be ideal to perhaps cite some guidelines, even recent ones, given the updates created for COVID, so that we have something "fresh" and up to date.

A paragraph was added (line 35-41) to the introduction with regard to current COIVD 19 treatment guidelines, as follows:

Recent data regarding respiratory support for COIVD-19 patients demonstrated lower risk of tracheal intubation in patients who were treated with continuous positive airway pressure (CPAP)(13), or with high flow nasal canula (HFNC)(14), both compared with low flow nasal cannula. No change was noted in mortality, nor in ventilator free days. Thus it seems that initial treatment with either CPAP or HFNC is acceptable(15), as reflected in current guidelines(16). However, a patient might deteriorate to IMV, which

4. Line 46 - 66 The authors in this point describe their work done previously. I did not find the need for this full digression on already published work, describing how the calculation is performed and how the prediction is developed. Ideally it would suffice to put the description much more shortened and succinct and then place the appropriate reference. Such a descriptive part should be included in the methods, in a dedicated section with respect to the predictive decision algorithm.

The paragraph describing our previous work (original lines 46-66) was shortened. Most of it was transferred into the methods section (line 91-99).

5. Rather, it would also be appropriate to describe and expand a little on the first part with respect to COVID respiratory failure or not, briefly, and describing any additional decision-making models, in addition to those described, or expanding a little on those already correctly cited.

The relevant paragraph was edited to briefly expand a little on the mentioned decision-making models as follows:

During COIVD-19 pandemic, other machine learning models were developed. Several models used a “snap-shot” of several factors (including comorbidities, vital signs, laboratory results, imaging results) taken at the emergency department or upon ICU admission. The models aim to predict deterioration to IMV, ICU admission, and/or mortality based on the “snap-shot” taken. In most of the models the prediction relates to the patients admission duration. AUC of the models ranges between 0.68-0.94(19–24).

Methods

6. In this section I find much confusion and lack of clarity in the development of the section. This section should perhaps be the most important section of the paper because it should describe the work done step by step, classifying it and clarifying all the variables. Instead, I find much difficulty in expression and a lack of clarity in the exposition and statement of the steps taken.

7. First, the inclusion criteria for which patient data were taken should be clarified.The authors state that they included all patients with COVID-19 infection. Were there no exclusions whatsoever in the patients? Precisely all patients were included. Were DNR patients not admitted? Or were they included in the data as well? The authors state that they included all hypoxemic patients by COVID-19 defining them as patients with an SpO2<88% or PaO2<60mmHg admitted for more than 48 hours. But did these patients have oxygen support? Where were they hospitalized? How were they being cared for from the respiratory point of view?

Inclusion and exclusion criteria were added, including reference to DNI/DNR patients.

A paragraph generally describing COVID-19 patients was added (lines 76-86) as follows:

We included all COVID-19 patients who were admitted to Rabin Medical Center hospital between March 1st 2021 and February 23rd 2022 who had hypoxemia (defined as pulse oximetry<88% or PaO2<60mmHg) and who were hospitalized for more than 48 hours. DNR/DNI patients were included, as the algorithm predicts the medical need in ventilation, but serves only as a support decision tool. Ventilated patients upon arrival (whether chronically ventilated or patients who were ventilated by the EMS) were excluded. Most of these patients were admitted to COVID-19 dedicated internal medicine wards. A small percentage were admitted to the ICU. Patients were treated based on COVID-19 treatment guidelines, mainly with steroids and anti-viral drugs as appropriate. Oxygen support was given to hypoxemic patients, mainly with low flow or high flow nasal canula, until an improvement in the hypoxemia was noted.

8. It is important to divide this section into subsections, so precisely to make the work clearer and more structured.

Subsections heading were added: Study population, Internal Validation of the Prediction model, Categorized model development, Statistical analysis.

9. A subsection should be the prediction model, with the part included in the Introduction and with the related calculations that were performed.

The paragraph were transferred from the introduction, and shortened a bit.

10. Another sub-section should also be all the subcategories created during the analysis.

Subsections heading were added: Study population, Internal Validation of the Prediction model, Categorized model development, Statistical analysis.

11. The chapter of the statistical analysis done with the results is missing. To be included within the work.

Statistical analysis section was added, as follows (lines 154-160):

All statistical analysis was done using Python [sklearn.metrics, scipy.stats]. For the internal validation positive and negative predictions were compared with the actual endpoint of each patient, whether ventilated or not. For the categorized model, positive and negative prediction were compared with the actual endpoint of each patient, whether ventilated/dead or not. Based on all observations, we constructed the confusion matrix, and calculated sensitivity, specificity, precision, and recall. This data allowed drawing an ROC, and its AUC was calculated. AUC p-values calculated using the one-sided Wilcoxon test comparing the ROC with the null hypothesis of the diagonal curve(29)

12. Line 116-119 I don't think it is very correct and appropriate to put unventilated and deceased patients in the ventilated category. They are patients with another category and other patients. They should not be included there. Or the authors should amply justify, perhaps with evidence or other references that they also made this inclusion and justify it properly.

The paragraph was edited. An explanation for including mortality of unventilated patients was added, and also a short review of machine learning models reported regarding this specific point, as follows (lines 141-151):

Since the algorithm is developed as a supporting decision tool, for this categorized model we counted patients who were not ventilated but died during the hospitalization as if they were ventilated ( as it is possible that ventilation was not done due to futility, or a prior patient and/or family preferences). Practically meaning, we aim to develop the algorithm to predict ventilation per se, leaving the decision whether to ventilate or not, to the physician discern based on the clinical scenario. Of the mentioned machine learning algorithms for COVID-19 patients, Gupta et al developed a prediction model for a composite outcome including mortality and respiratory deterioration(20). We could not find in other deterioration model descriptions a commentary to the specific situation of mortality without mechanical ventilation, nor a commentary to DNI/DNR status(18,19,23–26). Interestingly, also the mortality prediction models, do not mention DNI/DNR status(21,27).

Results

13. Figure 2

In the figure it would be important and more precise to also include the significance p-value as well as AUC.

P-values were added to the figure.

14. Table 2

Completely missing units of measurement.

Abbreviations used (ex. MIMIC, etc.) should be included in all tables.

Abbreviations and units of measurement were added to all tables.

Discussion

15. The discussion the authors undertake is correct. However, a structural problem is present. The discussion should be a comparison with the present literature. How come the authors, except at the beginning, do not compare with the literature? Even at moments, after the description and inferences made about their results, they should compare them with the present literature and assess whether they are overlapping, similar, probably similar, or completely different. This work is hardly developed in this section of the paper.

Discussion paragraphs were reedited. Comparison to current literature and comparison to current work was added.
